# ANOCRITICISM AND THE PERFORMANCE OF MASCULINITY IN OLDER MEN

**DOI:** 10.1093/geroni/igac059.1490

**Published:** 2022-12-20

**Authors:** Kate de Medeiros

**Affiliations:** Miami University, Oxford, Ohio, United States

## Abstract

While anocriticism is a feminist framework often applied in the context of literary gerontology, this paper uses an anocriticism approach to consider how hegemonic masculinities and old age reveal a separation between cultural stereotypes of age and chronological age. Narratives from 5 men aged 65 and over, who participated in a qualitative study on everyday attributions of depression, were analyzed using an anocriticism framework. Results revealed that participants both claimed and contested their chronological age as an identity marker. Of particular note were claims related to the stereotype of loss of vigor in later life, with each man making a clear point to affirm his virility and sexual prowess independent of other interview questions. Overall, findings point to an important way to read interview data through an anocritical lens, paying particular attention to ways that power are questions and performed in relation to age.

